# Emerging Developments in Microbiome and Microglia Research: Implications for Neurodevelopmental Disorders

**DOI:** 10.3389/fimmu.2018.01993

**Published:** 2018-09-03

**Authors:** Yeonwoo Lebovitz, Veronica M. Ringel-Scaia, Irving C. Allen, Michelle H. Theus

**Affiliations:** ^1^Graduate Program in Translational Biology, Medicine, and Health, Virginia Tech, Blacksburg, VA, United States; ^2^Department of Biomedical Sciences and Pathobiology, Virginia-Maryland College of Veterinary Medicine, Blacksburg, VA, United States; ^3^Department of Basic Science Education, Virginia Tech Carilion School of Medicine, Roanoke, VA, United States; ^4^Center for Regenerative Medicine, Virginia-Maryland College of Veterinary Medicine, Blacksburg, VA, United States

**Keywords:** autism, bacterial metabolites, gut bacteria, lactobacillus, microbiota, neurodevelopment, neuroimmune

## Abstract

From immunology to neuroscience, interactions between the microbiome and host are increasingly appreciated as potent drivers of health and disease. Epidemiological studies previously identified compelling correlations between perinatal microbiome insults and neurobehavioral outcomes, the mechanistic details of which are just beginning to take shape thanks to germ-free and antibiotics-based animal models. This review summarizes parallel developments from clinical and preclinical research that suggest neuroactive roles for gut bacteria and their metabolites. We also examine the nascent field of microbiome-microglia crosstalk research, which includes pharmacological and genetic strategies to inform functional capabilities of microglia in response to microbial programming. Finally, we address an emerging hypothesis behind neurodevelopmental disorders, which implicates microbiome dysbiosis in the atypical programming of neuroimmune cells, namely microglia.

## Introduction

The various microbial ecosystems (“microbiota”) and their component genes (“microbiomes”) existing on and within the host body are increasingly recognized as significant contributors to a functional host immune system. Commensal microbiota consist of bacteria, fungi, viruses, and other microorganisms that make up distinct microbial ecologies of various host systems, such as the gastrointestinal tract, skin, mouth, and genitourinary tract (1). Within each physiological niche, microbial compositions can vary widely according to the environment and mutualistic functions that they may serve in conjunction with the host (2). In the gut, where microbial density is the highest, some of these functions include forming a physical barrier against infection by pathogenic microbes, acting as a bioreactor for digestion and nutrient absorption, and sensitizing the host immune system (3). The latter interaction is especially critical in early development, as the colonization of the infant gut with an initial inoculum of maternal microbiota primes the neonatal peripheral immune system (4, 5).

Given the wealth of evidence depicting microbiota as a driver of early peripheral immunity, an important follow-up question remains as to whether the microbiome may also drive immune development in the brain. The neuroimmune system, primarily comprised of glial cells, is distinct from the peripheral immune system in part due to anatomical barriers and developmental sequence. Microglia, the resident macrophage-like cells in the brain, play an especially critical role in neurodevelopment through their numerous functions in patterning and wiring of the maturing brain. Accordingly, recent studies on neurodevelopmental disorders, such as autism spectrum disorders (ASD), Rett syndrome, and schizophrenia, whose complex pathologies include neuronal and synaptic dysfunction, suggest improper microglial activity as a contributor to these disorders' neurobiological and behavioral outcomes (6–8). Interestingly, additional studies suggest that microglia, not unlike peripheral macrophages, may be susceptible to microbiome changes (9, 10). Altogether, the demonstration of microbial influence on brain function via microglial mediators raises the possibility that manipulation of microbe-immune crosstalk represents a promising strategy for treating neurological diseases.

## Microbiome and neurodevelopment

A growing number of studies pointing to distinct gut microbiome profiles among psychiatric patient populations allude to microbiota as an important corollary of disease pathology (11–14). The purported mechanisms for microbial linkages to aberrant neurobehavioral outcomes are broadly considered to be due to impaired gut-brain communication, including but not limited to those cause by cytokine imbalance, vagal nerve signaling, and hypothalamic-pituitary-adrenal (HPA) axis responses (15–17).

**Figure 1:**
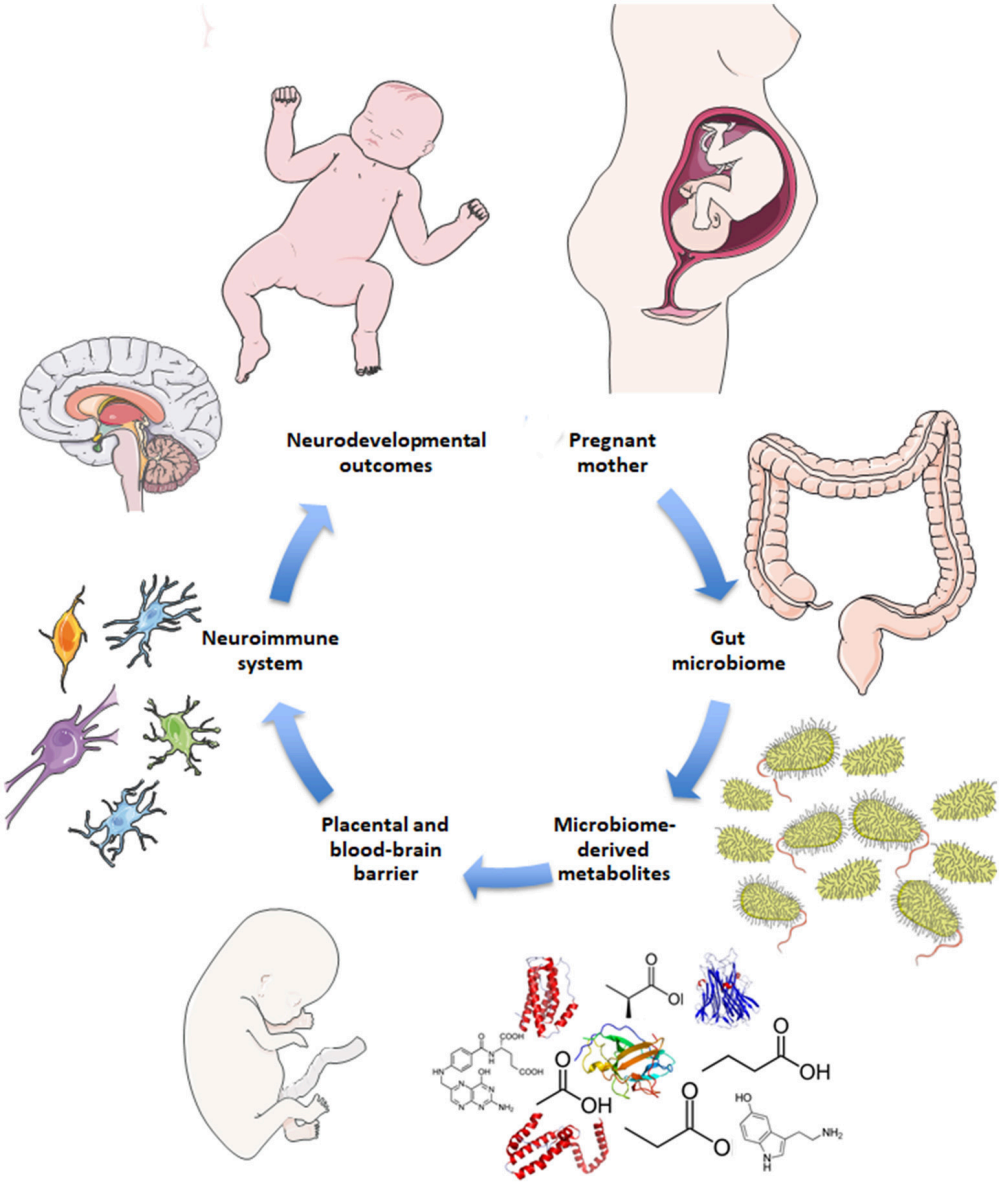
Schematic for maternal microbiome influence on neurodevelopment. Current hypotheses propose disruptions to the maternal gut microbiome during pregnancy, such as antibiotic use, lead to altered gut microbial communities and subsequently altered levels of microbe-derived metabolites and impaired immune signaling. Microbial metabolites include neurotransmitters, neuropeptides, and short-chain fatty acids that are small enough to bypass the placental and fetal blood-brain barriers. Microbial metabolites may serve neuroactive roles through immune priming interactions with microglia in the fetal brain to potentially drive neurodevelopmental changes and behavioral outcomes later in life.

Disruptions to gut microbiota are also implicated in aberrant neurodevelopmental outcomes (18–20). Here, the impaired gut-brain pathways are extrapolated to include both mother and fetus, i.e., the maternal gut-fetal brain axis. Permeability of maternal gut epithelium, the placental barrier, and fetal blood-brain barrier are potential factors of maternal gut-fetal brain communication, as are neuroactive microbial metabolites that are small enough to bypass these barriers (5, 21). The conceptual basis for how the maternal microbiome may drive offspring neurodevelopment is illustrated in Figure 1. Maternal skin and vaginal microbiota play a critical role in seeding the infant microbiota and were shown to contribute equally to various body site taxa in infants up to 6 weeks of age in vaginal deliveries and cesarean deliveries accompanied by active labor (22). In addition, amniotic fluid, placenta, and umbilical cord blood possess their own niche microbiomes, although the manner and extent to which these microbial communities communicate with the mother or fetus are not yet clear (23).

**Figure 2:**
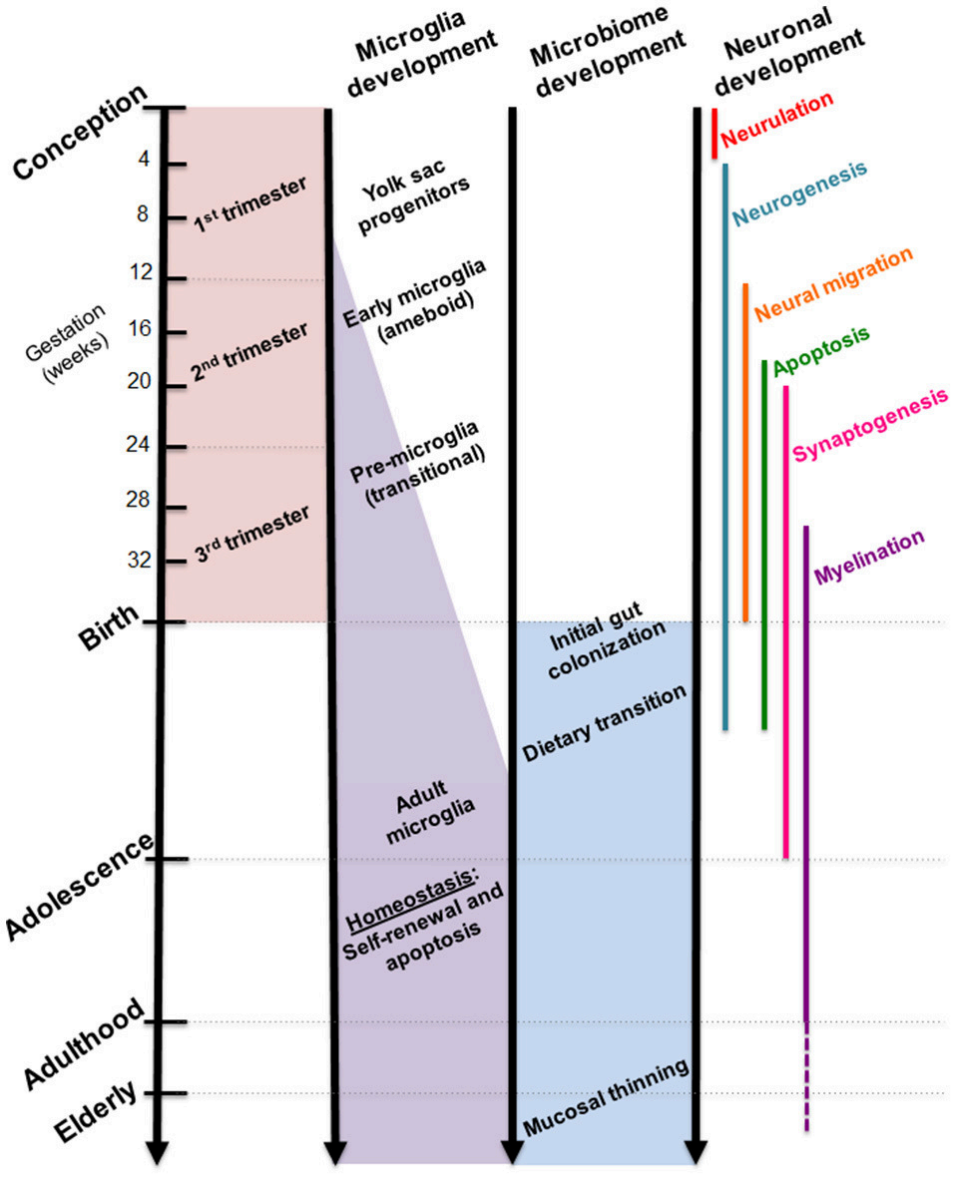
Comparative timelines for human microglia, gut microbiome, and neuronal development. Critical stages in brain development coincide with infant gut colonization to suggest maternal microbiome may serve as an important inoculum in priming the neuroimmune system.

While the gut microbiome is constantly evolving in response to dietary and environmental changes, longitudinal sampling of infant stool during the first 3 years of life demonstrated resiliency in its ability to return to original homeostatic conditions following short periods of antibiotic usage (24). Additional studies suggest that the overall composition of the gut microbiome may remain stable across multiple decades of life (25). Interestingly, developmental shifts in gut microbial composition align with milestones in brain development, such as neuronal migration and proliferation, myelination, and synaptic pruning (Figure 2). Although these developmental correlations do not necessarily indicate a causal relationship, strong evidence from experimental models using germ-free (GF) and antibiotic-treated rodents showed that the complete absence or severe reduction of gut microbiota, respectively, resulted in altered brain chemistry, transcriptional changes, and atypical behaviors compared to controls (18, 26, 27).

Nonetheless, large-scale epidemiological studies highlight compelling correlations between certain microbiome-modifying pregnancy events and subsequent diagnoses of neurodevelopmental disorders in children. Maternal infection and antibiotic use during pregnancy are often highlighted as potential risk factors for ASD (28–30). Of the former, meta-analysis of studies reporting ASD risk of maternal infections resulted in significant associations with bacterial infections during second and third trimesters. The odds of subsequent ASD diagnosis were slightly greater than those reported for viral infections, but not as high as any maternal infection combined with a hospital visit (31). Such results also comport with numerous animal studies that used viral or bacterial components to elicit maternal immune activation and resulted in broad-based ASD- and schizophrenia-like phenotypes in the offspring, including neuroinflammation, dysregulated neural circuitry, behavioral deficits, and gene expression changes (32–36). Within the context of the mother-child dyad, these findings suggest that acute, immune insults as a result of microbial dysbiosis during pregnancy may be more influential on neurodevelopmental outcomes than chronic conditions that mainly affect the mother alone.

### Gut bacteria

Gastrointestinal issues are a common comorbidity in ASD, which remains a factor even when considering non-autistic sibling controls (37). Comparative gut microbial profiling studies in ASD suggest that, in the absence of a pathogenic infection, the lack of commensal microbes and/or microbial communities found in neurotypical counterparts may contribute to adverse health outcomes. Pyrosequencing of fecal bacteria DNA in children diagnosed with ASD determined lower abundance of gut bacteria species known to ferment complex carbohydrates, such as *Prevotella, Coprococcus*, and *Veillonellaceae* compared to non-autistic children (38). Meanwhile, other studies reported increased abundance of *Bacteroides, Ruminococcus, and Sutterella* in autistic children compared to controls (39, 40). A recent open-labeled clinical study showed that fecal microbiota transplantation resulted in mitigation of both gastrointestinal and behavioral symptoms in autistic children that corresponded with increased diversity of gut microbiota and increased abundance of previously low populations, such as *Prevotella* (41). Accordingly, targeted communities of commensal gut microbiota are currently under investigation as possible catalysts for gut-brain signaling. At present, research on these microbes are bacteria-specific and frequently coincide with research on probiotics. Of clinical interest are bacterial species found in maternal microflora during pregnancy and in the neonatal gut, such as *Lactobacillus, Bifidobacterium*, and *Bacteroides*, whose presence represent homeostatic conditions during healthy development (42).

*Lactobacillus* is a key component of a complete and diverse gut microbiome and represents a genus of bacteria naturally found in the gut of healthy mammals. The human vaginal tract microbiome is also primarily dominated by *Lactobacillus* spp. followed by anaerobic species from *Prevotella* and *Sneathia* spp. (42). During pregnancy, the vaginal microbiome undergoes remodeling that results in reduced diversity, increased stability, and enrichment of *Lactobacilli*. This is thought to protect against pathogenic infection through increased lactic acid production and decreased pH levels (43, 44). Parallel sampling of maternal and neonatal microbiota showed that the gut microbiota of vaginally-delivered infants reflects bacterial species found in the maternal gut microbiome, whereas cesarean-delivered infants were more likely to harbor bacteria from maternal skin microbiome, e.g., *Staphylococcus, Corynebacterium*, and *Propionibacterium* spp. (42). Meanwhile, the use of intrapartum antibiotics resulted in infant gut dysbiosis at 3 months and 12 months of age regardless of the mode of delivery (45).

As a commensal microbe, *Lactobacilli* in the gastrointestinal tract confer beneficial effects to digestion through lactic acid fermentation of foods (46) and prime immune cells via interactions with leukocytes in mesenteric lymph nodes and/or via dendritic cell sampling of gut lumen contents (47–49). The influence on immune system priming has been shown to have significant effects in biological and behavioral outcomes. For example, changes to HPA axis signaling and reduced corticosterone levels were observed in mice following oral administration with various *Lactobacilli* strains, such as *L. rhamnosus*, (16). Similarly, *L. reuteri* represents another well-studied strain with reports of restoration to ventral tegmental area synaptic plasticity and oxytocin production in mice born to dams on a high fat diet (50). Meanwhile, administration of *L. helveticus* resulted in decreased levels of inflammatory cytokines and improved performance in spatial memory and anxiety-related behavior tasks in IL-10^−/−^ mice (51).

Given the broad evidence base supporting the molecular and physiological impact of *Lactobacillus* spp., the continued focus on this genus of bacteria is not surprising. Nonetheless, other bacteria, such as *Bifidobacteria* and *Bacteroides*, have also demonstrated ability to regulate immune response and behavior. For example, oral feeding of *Bacteroides fragilis* to weaned mice in a maternal immune activation model of autism resulted in the recovery of gut barrier proteins, Claudin-5 and-8, and rescue of anxiety-like and stereotypic behaviors (19). These findings suggest that bacterial functionality, such as the ability to sensitize immune cells or produce bioactive metabolites, may be a better indicator of gut-brain interaction than mere taxonomy.

### Gut bacterial metabolites

The discovery of penicillin by Alexander Fleming popularized the notion that byproducts of microbial metabolism could serve as potent chemicals (52). These metabolic byproducts, or metabolites, range broadly in terms of their molecular assembly and function. They can act as quorum sensing molecules, energy substrates, or even competitive antimicrobials against other microbes (53–55). Microbial metabolism is also one of the critical functions of the gut microbiome in maintaining host health; the mammalian digestive system is incapable of extracting many key nutrients, such as vitamins, amino acids, and energy, from diet and relies on commensal gut microbes for these tasks (56).

The most abundant products of gut bacterial metabolism are short-chain fatty acids (SCFAs), which result from bacterial fermentation of complex carbohydrates and proteins in the colon. SCFAs refer to fatty acids consisting of one to six carbon atoms, but predominantly consist of acetic acid, butyric acid, and propionic acid in the mammalian gut (57). Of the three, acetic acid (anion: acetate) makes up the largest portion of SCFA distribution in the colon, where it readily enters the circulatory system to act as a vasodilator or energy substrate for peripheral tissues (58). Radiolabeled colonic acetate has been shown to pass the blood-brain barrier to serve as an energy substrate for astrocytes, but also to preferentially accumulate in the hypothalamus where it is converted to acetyl-CoA leading to downstream suppression of appetite-related hormones, Neuropeptide Y (NPY) and agouti-related peptide (AgRP) (59, 60). Butyric acid (anion: butyrate) is an important energy substrate for colonocytes and a well-documented histone deacetylase (HDAC) inhibitor with pharmaceutical potential for neurodegenerative diseases (61, 62). Interestingly, exposing microglia to sodium butyrate *in vitro* resulted in differential inflammatory responses wherein rat primary cells, hippocampal slice cultures, and neural co-cultures (consisting of microglia, astrocytes, and neurons) resulted in an anti-inflammatory effect against LPS, but cultured murine N9 microglial cells elicited a pro-inflammatory response (63). Propionic acid (anion: propionate) appears to be the only SCFA to demonstrate adverse effects in the brain, as direct intracerebroventricular injection of propionic acid in rats yielded a wide range of autism-like neurobehavioral changes, including repetitive motion and increased markers for astrocyte and microglia immunoreactivity (GFAP and CD68, respectively) (64).

While SCFAs' best known functions are to serve as fuel for colonocytes and regulators of host metabolism, recent investigations revealed that SCFAs directly interact with the nervous system via G protein-coupled receptors, GPR41 and GPR43 (or free fatty acid receptor 3 [FFAR3] and FFAR2, respectively) (65, 66). Previously deemed “orphan” receptors, GPR41 and GPR43 are now understood to be expressed broadly on host tissues and immune cells and are involved in the resolution of inflammatory responses (67–69). Within the brain, GPR41 is expressed at low levels in the cerebral cortex, hippocampus, caudate, and cerebellum and preferentially binds to butyrate and propionate, whereas GPR43 is expressed at moderate levels in the caudate and preferentially binds to acetate and propionate (67, 70). Much remains to be discovered about the signaling mechanisms of these receptors, but emerging studies point to downstream activation of immune responses, such as IgA promotion and inhibition of NF-κB pathway, that are specifically triggered according to the type of metabolite ligand and location of the receptor (71, 72).

## Microbiome and microglia

Microglia are primarily recognized as the resident macrophage-like cells in the brain that serve as first-responders to pathogens, apoptotic cells, and debris with the ability to secrete soluble factors that modulate inflammatory responses. Recent studies paint a more complex portrayal of these highly-motile glial cells, which have since been found ubiquitously throughout the central nervous system, including the spinal cord, with region-specific phenotypes (73–75). Microglia originate as yolk sac progenitors (Figure 2) and are purported to migrate to the brain during early prenatal development, as demonstrated through single-cell RNAseq studies (10, 76). Perhaps appropriate given their presence in the embryo, microglia serve numerous critical functions in wiring and patterning of the developing brain. Through the production of neurotrophic factors, microglia contribute to neurogenesis and guidance of sprouting vessels, as well as phagocytosing synapses and shaping neuronal circuitry (77, 78). The stepwise processes in which these actions occur are not yet fully clear, although recent studies form a widening picture of microglial contribution to the proper maturation of the brain.

Current hypotheses surrounding the underlying etiology of neurodevelopmental disorders focus on microglia's dual immune and trophic capacities. Neuroimaging studies of ASD patients showed hypermyelination in both left and right medial frontal cortex, hypomyelination of the left temporo-parietal junction, and decreased local and long-range functional connectivity (79, 80). These findings are supported by murine models of ASD, whereby impediments to microglial functions resulted in under-pruning of synapses, hypermyelination of the prefrontal cortex, and reduced long-range functional brain connectivity (81–83). In contrast, rodent models of schizophrenia allude to an over-pruning effect of dysfunctional microglia wherein pharmaceutical intervention using minocycline resulted in stalled engulfment activity and rescued behavioral deficits (8, 84, 85). Genomic analysis of psychiatric disorders indicated upregulation of astrocyte-related genes and downregulation of neuronal/microglia-related genes across ASD, schizophrenia, and bipolar disorder to further propose a shared susceptibility in neuroimmune-specific gene networks (86).

In the gastrointestinal tract, microbiota and associated metabolites have been shown to elicit both pro- and anti-inflammatory peripheral immune cell responses and to control cellular proliferation and epithelial barrier integrity (47, 87–89). Evidence of increased blood-brain barrier disruption, altered microglia morphology, and increased microglia density in GF mice suggests that the microbiome may have a similar influence on the brain (9, 10, 21). However, contrary to expectations, gene expression analysis of postmortem cerebral cortex and cerebellum tissues from ASD patients showed upregulation of genes associated with barrier proteins (e.g., Claudin-5 and TRiC) compared to controls; the same analysis of small intestine duodenal tissue revealed decreased expression of barrier protein-associated genes (90). Together, these data suggest that the epithelial cell barrier integrity in the gut is compromised, but barrier protein expression in the brain is increased in ASD.

The microbiome is also implicated in the neuroinflammation hypothesis underlying the ontogeny of neurodevelopmental disorders. With respect to ASD, anatomical evidence of unusual and sustained brain overgrowth in autistic children first alluded to improper cellular responses wherein the hyperproliferative stage of early neurodevelopment continues unchecked and unresolved (91, 92). Thus, the neuroinflammation hypothesis stipulates neonatal microbial dysbiosis leads to improper priming of the immune system, which leads to reduced synaptic pruning and brain overgrowth in ASD. Paradoxically, immunohistochemical and cytokine profiling of brain tissue and cerebrospinal fluid samples from ASD patients showed abundance of activated microglia and increased expression of macrophage chemoattractant factor-1 (MCP-1) and tumor growth factor-β1 (TGF- β1) in cerebral cortex, white matter, and cerebellum (93). Additional studies of ASD postmortem brain tissue also depicted decreased numbers of ramified, or resting state, microglia in gray and white matter and increased primed, or activated, microglia in gray matter of ASD brains compared to control samples (7, 94). The exact mechanism by which activated microglia neglect to phagocytose excess myelin, synapses and/or neurons, and whether this is mediated by a microbiome-immune crosstalk, is yet unknown.

Increasing evidence suggests the microbiome plays a contributing role in the function of microglia in neurodevelopment. A number of models were used to evaluate cause and effect through a reductive process that includes the use of GF models. Erny, et al., found that compared to specific-pathogen free (SPF) mice, GF microglia had marked differences in mRNA expression profiles, including a reduction in genes associated with cell activation and immune signal transduction (9). While no gross or histologic abnormalities were observed in the central nervous system, GF microglia displayed an immature morphology, reduced capacity to respond to a viral infection challenge, and significant reduction in expression of regulators of microglia cell proliferation, differentiation, activation, and transformation (9). Additionally, microglia of antibiotic-treated and *Ffar2*^−/−^ mice that do not express the receptor gene for SCFA-binding also resembled a GF phenotype, which could be rescued by oral feeding of SCFAs in the former group but not the latter (9). Thus, bacteria-derived metabolites likely play a necessary and sufficient role in proper microglia development, although the mechanism(s) regarding how microbiota influence microglia-neuron interactions during development and the behavioral consequences that ensue following dysbiosis remain elusive.

## Conclusion

The numerous microbiome-related studies across disparate scientific disciplines agree on a prevailing hypothesis that the microbiome is capable of communicating via immune, metabolic, and endocrine signals to modulate brain health and disease. The current evidence base from both human and animal studies to support this hypothesis, however, are largely correlational without definitive understanding of cause. The mechanisms by which the microbiome asserts microglial changes during neurodevelopment are also not yet known, although transgenic mice and single cell sequencing methods continue to inform molecular processes that are likely to be involved. At present, the majority of microbiome-related studies are conducted in adult mice and assume a linear direction of influence from microbiota to the host. In recognition of the microbiome's role in neurodevelopmental processes, future studies should include appropriate experimental models that address the maternal gut-fetal gut axis as well as the possibility of multidirectional signaling pathways.

## Author contributions

YL, VR-S, IA, and MT contributed to the manuscript preparation and writing. YL and VR-S generated figures. IA and MT conducted final editing and review of the manuscript.

### Conflict of interest statement

The authors declare that the research was conducted in the absence of any commercial or financial relationships that could be construed as a potential conflict of interest.
